# Multimodal Free-Water Imaging Links Cardiometabolic Risk to Periarterial Dysfunction and Amyloid Accumulation in Early Alzheimer’s

**DOI:** 10.21203/rs.3.rs-9374985/v1

**Published:** 2026-05-07

**Authors:** Yaqiong Chai, Hedong Zhang, Andrew S. Kim, Quanhao Sun, Shuting Chen, Jongmok Ha, Kyung Wook Kang, Harmony Cen, Jung Won Shin, Gilsoon Park, Jiyeah Choi, Minji Kim, Neda Jahanshad, Paul M. Thompson, Helena C. Chui, Danny JJ Wang, Diane C. Lim, Eun Yeon Joo, Arthur W. Toga, Hosung Kim

**Affiliations:** Keck School of Medicine of University of Southern California; Keck School of Medicine of University of Southern California; Keck School of Medicine of University of Southern California; Keck School of Medicine of University of Southern California; Keck School of Medicine of University of Southern California; Samsung Medical Center, Sungkyunkwan University School of Medicine; Chonnam National University Hospital; Keck School of Medicine of University of Southern California; Samsung Medical Center, Sungkyunkwan University School of Medicine; Keck School of Medicine of University of Southern California; Keck School of Medicine of University of Southern California; Keck School of Medicine of University of Southern California; Keck School of Medicine of University of Southern California; Keck School of Medicine of University of Southern California; University of Southern California; Keck School of Medicine of University of Southern California; University of Miami; Samsung Medical Center, Sungkyunkwan University School of Medicine; Keck School of Medicine of University of Southern California; Keck School of Medicine of University of Southern California

## Abstract

The brain’s waste-clearance (glymphatic) system removes metabolic byproducts via periarterial influx, interstitial exchange, and perivenous efflux. Although dysfunction is implicated in Alzheimer’s disease (AD), current imaging markers emphasize perivenous changes and may overlook earlier periarterial impairment. We developed a diffusion MRI framework to quantify periarterial fluid mobility, white matter free water, and perivenous integrity, and applied it to 546 cognitively normal adults (HCP-Aging) and 173 participants across the AD spectrum (ADNI). Periarterial mobility was reduced with higher cardiometabolic risk and amyloid positivity, particularly in AD-vulnerable regions. Free water increased with aging and metabolic burden, whereas perivenous dysfunction was most pronounced in AD. Combined measures predicted amyloid positivity and cognitive impairment (AUC = 0.82). Mediation analyses showed that blood pressure influenced cognition through periarterial dysfunction and amyloid burden. These findings support a staged, compartment-specific trajectory of glymphatic dysfunction, with early periarterial impairment representing a potential biomarker and therapeutic target.

## Introduction

Alzheimer’s disease (AD) is defined by progressive impairment of memory and cognitive function and by the pathological accumulation of amyloid-β (Aβ) plaques and tau neurofibrillary tangles, hallmarks central to disease pathogenesis. Emerging evidence indicates that impaired clearance of Aβ via the glymphatic system significantly contributes to AD onset and progression^1,2^. Insufficient removal of tau aggregates may further accelerate cognitive decline^3^.

The glymphatic system supports brain waste clearance through three principal phases: cerebrospinal fluid (CSF) influx into periarterial spaces (pAs), bulk solute exchange with interstitial fluid (ISF) in brain parenchyma, and efflux via perivenous spaces (pVs) toward lymphatic drainage. Yet, the relative vulnerability and distinct contributions of these three components to protein clearance impairments in AD, especially in humans, remain poorly understood.

Current *in vivo* human imaging methods of the glymphatic system predominantly rely on diffusion tensor imaging along the perivascular space (DTI-ALPS)^4^, which quantifies water diffusivity within pVs near paraventricular white matter. While the ALPS index is significantly reduced in AD, its sensitivity to glymphatic dysfunction in early AD stages is limited^5^. Studies in individuals with mild cognitive impairment (MCI) or subjective cognitive decline have yielded inconsistent results, partly due to the ALPS index’s regional specificity. It primarily captures changes near the lateral ventricles and may miss early alterations in regions more vulnerable to initial amyloid pathology, such as the precuneus, or tau pathology, such as the entorhinal cortex^6^.

Additionally, emerging hypotheses propose distinct underlying mechanisms of glymphatic dysfunction across disease stages. For instance, early-stage impairment may stem from reduced periarterial CSF influx due to arterial stiffening and diminished pulsatility, resulting in pressure reversal, impaired solute clearance, and cerebral amyloid angiopathy. In contrast, disruptions detected by ALPS in the perivenous system may reflect downstream consequences of extensive parenchymal Aβ accumulation in later disease stages.

Moreover, the presence of other comorbidities may additively impair the glymphatic system (e.g., hypertensive arteriolosclerosis may decrease pulsatility in pAS, while obstructive sleep apnea or traumatic bleeding into the subarachnoid space may reduce CSF absorption in the pVs). For instance, pAs function, reliant on arterial compliance, may be more vulnerable to vascular risk factors such as hypertension, hyperlipidemia, diabetes and obesity^7,8^. Conversely, pVs dynamics are influenced by respiratory mechanics and may be adversely affected by sleep-disordered breathing, which commonly precedes cognitive impairment and may exacerbate glymphatic dysfunction^9^.

While increased free water volume fraction (FWVF) in white matter (WM) has been proposed as a potential imaging correlate of glymphatic dysfunction^10^, the field still lacks a direct, validated in vivo imaging framework that can evaluate water diffusivity across all three compartments of the glymphatic pathway. This creates a critical knowledge gap regarding glymphatic dysfunction mechanisms across various AD stages, age groups, and comorbidities.

To address this gap, we leveraged two complementary imaging cohorts: the Human Connectome Project in Aging (HCP-A) to examine glymphatic vulnerability to cardiometabolic risk factors and sleep in cognitively normal older adults^11^, and the Alzheimer’s Disease Neuroimaging Initiative (ADNI) to characterize glymphatic alterations associated with Aβ and tau pathology, clinical disease severity, and cognitive impairment^12^. This dual-cohort approach allowed us to evaluate both early vulnerability and AD-specific dysfunction in a stage-sensitive manner.

In this study, we set out to quantify glymphatic clearance across the three core compartments using multimodal MRI. Specifically, we aimed to: (1) identify glymphatic changes linked to aging, sleep, and cardiometabolic syndrome (CMS) in cognitively normal (CN) adults; (2) determine glymphatic components most strongly associated with Aβ and tau pathology; (3) characterize glymphatic disruption across CN, mild cognitive impairment (MCI), and AD cohorts; (4) evaluate the predictive value of glymphatic markers in classifying MCI or MCI/AD from CN individuals; and (5) map regional patterns of glymphatic dysfunction linked to aging and CMS, and AD progression.

## Results

### Study participants

For the analysis of free-water diffusivity using multi-shell DWI and bi-tensor modeling, we analyzed 579 cognitively normal subjects (age range: 36–100 years; 58.6 ± 14.1 years; 312 females) from the HCP-A Release 2.0 and 10 AD dementia patients, 68 MCI patients, and 95 CN controls from the ADNI-3 and ADNI-4 datasets (age range: 36–100 years; 76.5 ± 7.3 years; 78 females).

Initially, 687 subjects in HCP-A had MRI data available. However, 63 participants were excluded due to poor data quality following quality assurance protocols; 48 were excluded due to lack of the CMS and sleep data; 28 were excluded due to segmentation errors; and 2 were excluded due to anatomical abnormalities, resulting in 546 subjects from the HCP-A cohort in the present analysis. The demographic and clinical characteristics of the HCP-A and ADNI samples are summarized in Table 1 and Table 2, respectively.

### Multimodal MRI metrics to understand glymphatic function across the three compartments

We estimated the following complementary metrics: 1) pAs diffusivity, quantified using free-water axial diffusivity (FWAD) and additional diffusion parameters (radial diffusivity, mean diffusivity), along with pAs-FWVF; 2) WM-FWVF, reflecting parenchymal fluid accumulation and ISF–CSF exchange efficiency; and 3) DTI-ALPS index, reflecting perivenous efflux integrity.

Motivated by prior evidence that MRI-visible perivascular spaces are predominantly periarteriolar in nature^13^, we quantified pAs diffusivity within perivascular spaces segmented from T1-weighted MRI. Multi-shell DWI data were analyzed using tissue tensor imaging (TTI), an advanced bi-tensor approach that separates tissue and free-water components while allowing anisotropic diffusion in the free-water compartment^14–16^. In contrast to conventional free-water–eliminated DTI, TTI enables directional characterization of fluid diffusivity along CSF-filled conduits such as the pAs (**Supplementary Methods**).

**Figure 1:**
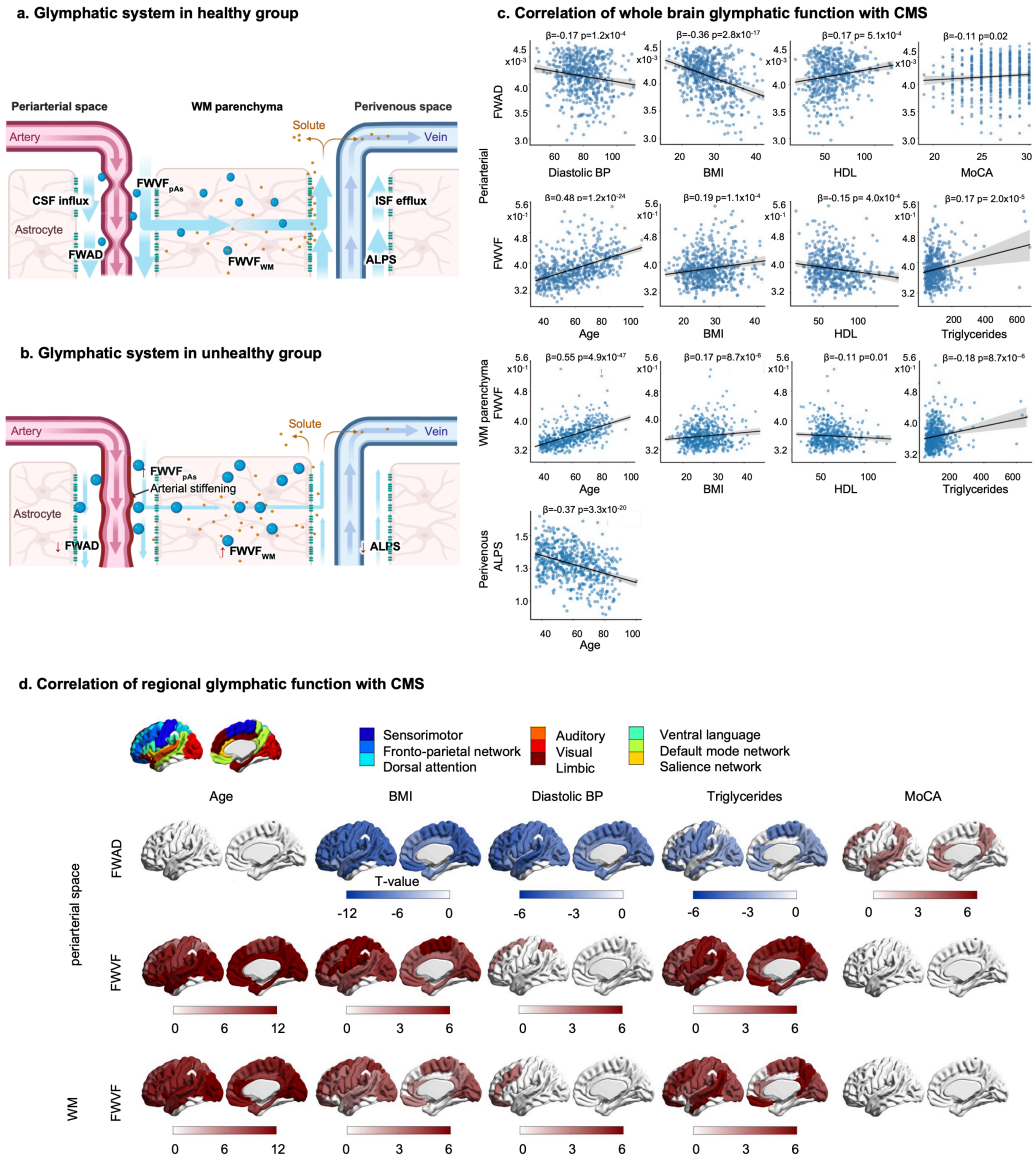
Conceptual framework and whole-brain associations of glymphatic MRI metrics. a. In healthy individuals, cerebrospinal fluid (CSF) flows along periarterial spaces (pAs), facilitating perivascular CSF influx into the white matter (WM) parenchyma. This enables efficient interstitial solute clearance through perivenous spaces (pVs) toward lymphatic vessels. Low free water volume fraction (FWVF) in both pAs and WM, along with preserved axial diffusivity (FWAD) and higher ALPS index, reflects intact glymphatic flow. b. In individuals with vascular or metabolic risk, arterial stiffening impairs periarterial CSF influx, reflected by reduced FWAD and increased FWVF in pAs. Elevated FWVF in WM impedes interstitial fluid (ISF) flow and CSF-ISF exchange. The diminished ALPS index indicates reduced ISF efflux in pVs. c. We analyzed whole brain fluid diffusion metrics for healthy subjects in HCP-Aging cohort. Whole brain pAs FWAD correlates with cardiometabolic syndrome (CMS) risk factors, and cognitive measure (MoCA). pAs- and WM-FWVF correlated with age and cardiometabolic syndrome (CMS) risk factors. ALPS correlates only with age. d. Regional correlation of pAs-FWAD and pAs- and WM-FWVF with age, CMS, and MoCA. All analyses were adjusted for age and sex, except the correlation with age (adjusted for sex only).

**Figure 2:**
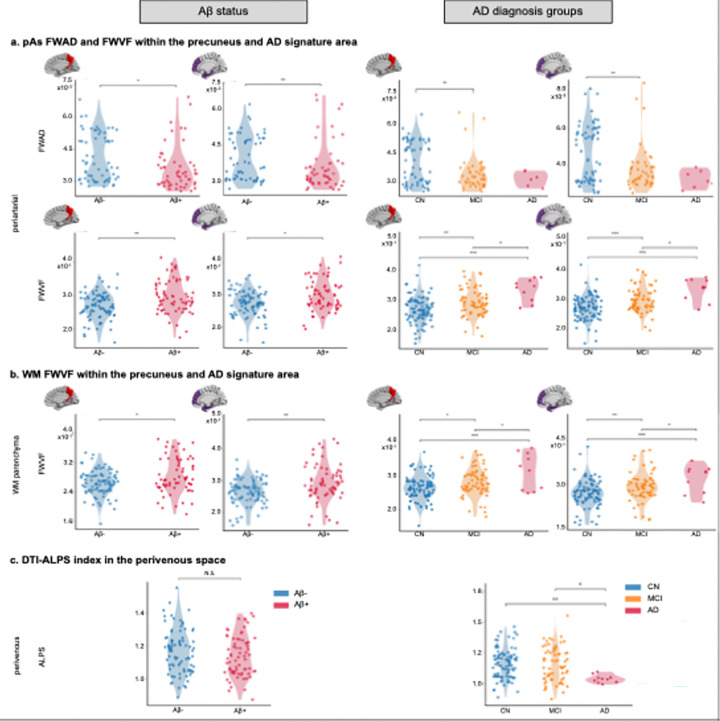
Group differences in glymphatic MRI measures across periarterial, white matter, and perivenous compartments. Violin plots show group-wise distributions of the measures: a. pAs-FWAD and FWVF within the precuneus and AD-signature regions; b. WM-FWVF within the precuneus and AD-signature regions; c. DTI-ALPS index in the perivenous space. Significant group differences were assessed using ANCOVA followed by Tukey’s HSD post hoc tests. Asterisks denote statistical significance (*p < 0.05, **p < 0.01, ***p < 0.001). All analyses were adjusted for adjusting for age, sex, education, and APOE carrier status. Abbreviations: pAs: periarterial spaces; WM: white matter; FWVF = free water volume fraction; FWAD = free water axial diffusivity; DTI-ALPS = DTI along perivascular space.

**Figure 3:**
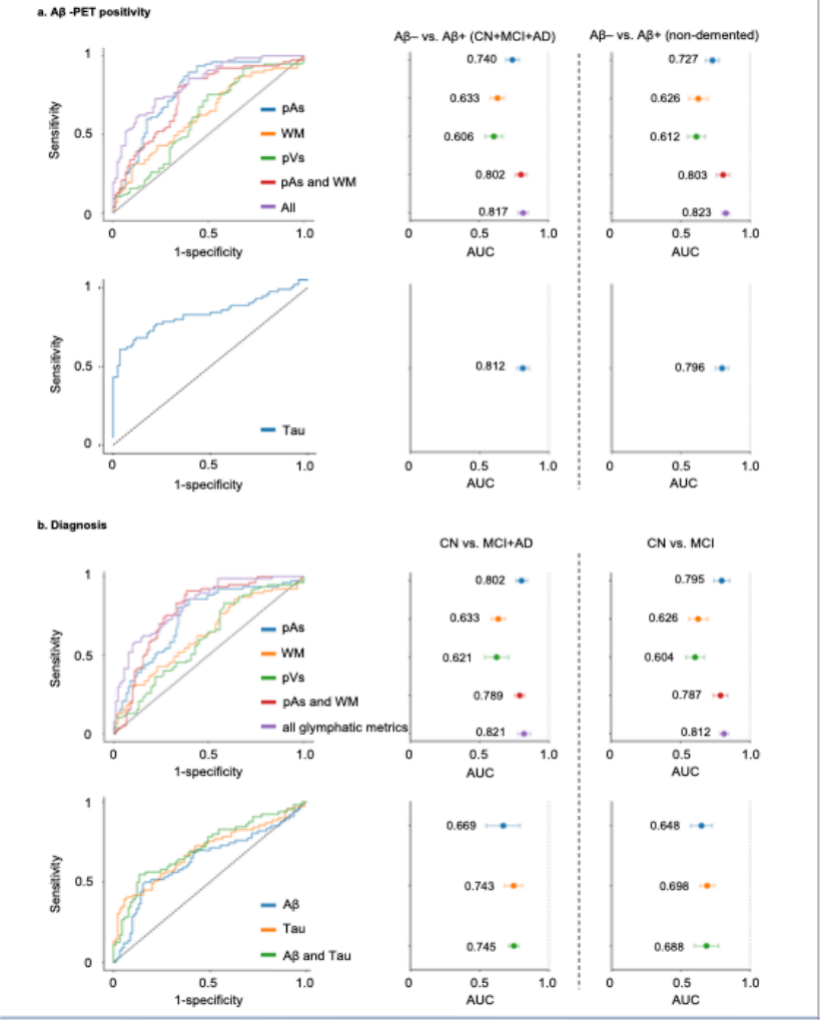
Predictive performance of glymphatic MRI metrics for Aβ positivity and diagnostic classification. Ten-fold stratified cross-validation was used for all models. a. Aβ-PET positivity classification. Receiver operating characteristic (ROC) curves and area-under-the-curve (AUC) values show the performance of glymphatic metrics (pAs, pVs, WM, pAs+WM, and combined features) to predict Aβ-PET positivity across all participants (CN + MCI + AD) and within the non-demented subgroup. Integrated models achieved the highest performance (AUC = 0.817–0.823), comparable to tau PET prediction (AUC = 0.796–0.812). b. Diagnostic classification. ROC curves and AUCs demonstrating performance of glymphatic metrics for distinguishing CN from MCI+AD and CN from MCI, compared with Aβ, tau, and combined PET-based models. The integrated glymphatic model outperformed Aβ or tau PET individually (AUC = 0.812–0.821 vs. 0.688–0.745).

**Figure 4:**
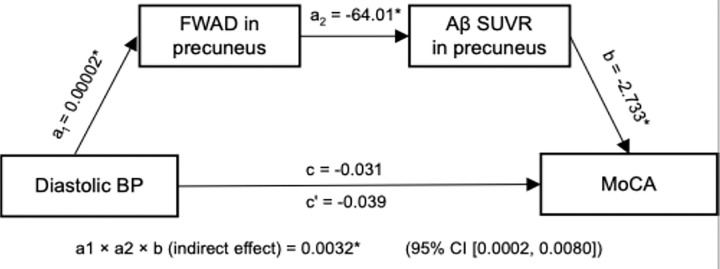
Mediation model of the effect of diastolic blood pressure (BP) on cognition (MoCA score) via precuneus periarterial microstructure and amyloid β burden (Aβ SUVR). Standardized path coefficients (β) are shown next to each arrow; * indicates p < 0.05. Diastolic BP positively predicted free water axial diffusivity (FWAD) in the precuneus (a_1_ = 0.00002, p < 0.05), which in turn was inversely associated with Aβ SUVR in the precuneus (a_2_ = −64.01, p < 0.05). Higher Aβ SUVR was linked to poorer cognition (b = −2.733, p < 0.05). The total effect of BP on MoCA (c = −0.031, p = ns) and the direct effect controlling for both mediators (c′ = −0.039, p = ns) are shown. The indirect (mediated) effect, calculated as a_1_ × a_2_ × b, was significant (0.0032*, 95% CI [0.0002, 0.0080]).

Figure 1 provides a conceptual illustration of glymphatic alterations in healthy versus pathological states based on our findings in the following sections (Fig. 2–4). In healthy brains, robust CSF mobility along the pAs (high FWAD), low FWVF in pAs and WM, and high pVs-ALPS indices signify effective glymphatic circulation. In contrast, pathological states exhibit diminished pAs FWAD (impaired influx), elevated FWVF (fluid retention and greater flow impedance), and reduced ALPS (impaired pVs-mediated efflux), consistent with progressive glymphatic failure (Fig. 1a–b).

To minimize partial-volume contamination in pAs diffusivity measurements, it was essential that the selected pAs segments be sufficiently large relative to the spatial resolution of the DWI data. By selecting the four largest pAs per region of interest (ROI), we observed that the mean pAs diameters in both HCP-A and ADNI cohorts exceeded the corresponding DWI voxel sizes (**Tables S1–S2**; HCP-A: pAs diameter = 1.7–2.2 mm vs. 1.5 mm isotropic DWI; ADNI: 2.7–3.0 mm vs. 2.0 mm isotropic DWI). We further validated that this choice provided optimal stability and sensitivity of associations with clinical variables compared with other choices of *n* largest pAs (see Methods and **Supplementary Fig. S1**), supporting the use of the four-largest-pAs threshold in subsequent analyses.

### Association of whole white matter glymphatic measures with aging, sleep, cognition, and CMS in HCP-A

Whole WM glymphatic measures were examined in healthy aging cohorts after adjusting for effects of age and sex. In the periarterial compartment (Fig. 1c; **Supplementary Fig. S2**), FWAD exhibited significant negative correlations with multiple CMS indicators, including body mass index (BMI; β = − 0.36, p = 2.8×10^−17^), systolic blood pressure (β = − 0.18, p = 1.0×10^− 4^), diastolic blood pressure (β = − 0.17, p = 1.2×10^− 4^), mean arterial pressure (MAP; β = − 0.19, p = 3.0×10^−5^), hemoglobin A1c (HbA1c; β = − 0.16, p = 5.1×10^− 4^), and fasting glucose (β = − 0.15, p = 0.001), while showing a positive correlation with high-density lipoprotein (HDL; β = 0.17, p = 5.1×10^− 4^). FWAD was not associated with age (p = 0.10). The effects of fasting glucose and blood pressure on pAs-FWAD were significant (p = 5.1×10^−6^ and 6.7×10^−4^, respectively), but after adding antihypertensive and diabetic medication use as covariates, these correlations were no longer significant (p = 0.236 and 0.968). On the other hand, FWVF increased with age (β = 0.48, p = 1.2×10^−24^), and was positively associated with BMI (β = 0.19, p = 1.2×10^− 4^) and triglycerides (β = 0.17, p = 2.0×10^− 5^), but negatively correlated with HDL (β = − 0.15, p = 1.1×10^− 4^). All p-values reported after false discovery rate correction.

After adjusting for age (but not sex), cognitive performance (MoCA) was positively correlated with FWAD (β = 0.11, p = 0.02). This relationship was observed in males (β = 0.11, p = 0.02) but not in females. No significant correlations between global pAs metrics and sleep quality (PSQI) were found.

Comparisons with other diffusivity metrics, including free-water radial diffusivity (FWRD), mean diffusivity (FWMD), and fractional anisotropy (FWFA) revealed similar directional trends but weaker associations (**Supplementary Fig. S3**). Fewer CMS factors were significantly correlated with these metrics, and their absolute β-values were consistently smaller than those for FWAD. Thus, FWAD was selected as the representative marker of glymphatic diffusivity for subsequent analyses.

In WM parenchyma, FWVF positively correlated with age (β = 0.55, p = 4.9×10^−47^), BMI (β = 0.17, p = 8.7×10^−6^), and triglyceride levels (β = 0.18, p = 8.7×10^−6^), and negatively with HDL (β = − 0.11, p = 0.01). No correlation with PSQI was found. This pattern was similar to that of FWVF in pAs.

Finally, the ALPS index, reflecting perivenous efflux, showed a robust negative association with age (β = − 0.37, p = 3.3×10^−20^) but no correlation with PSQI, MoCA, or CMS factors.

### Regional association of glymphatic metrics with aging, sleep, cognition, and CMS in HCP-A.

As we divided the entire WM into nine functional ROIs (see Methods), region-specific analyses (Fig. 1d) revealed differential spatial patterns of glymphatic vulnerability in pAs and WM parenchyma to CMS factors. Note that no regional pattern is available for pVs ALPS as this index is a single global measure.

Across nine ROIs, age showed widespread positive associations with FWVF in both pAs and WM parenchyma, spanning the frontoparietal (FPN), dorsal attention, ventral language, sensorimotor, auditory, visual, salience, default mode (DMN), and limbic networks (p < 0.05). No regional FWAD associations with age were detected.

Of the CMS variables, higher BMI was associated with lower FWAD and higher FWVF in pAs across all ROIs, paralleling the WM-FWVF pattern, though effects were most pronounced in the DMN, salience, sensorimotor, dorsal attention, and visual networks. Higher diastolic blood pressure was linked to widespread lower FWAD across networks and to higher FWVF restricted within the dorsal attention network (pAs) and FPN (WM).

Higher triglyceride levels were associated with lower pAs-FWAD in the sensorimotor, FPN, visual, and DMN regions, and with higher FWVF across all WM networks except the limbic region. Furthermore, as in **Supplementary Fig. S4**, lower HDL cholesterol correlated with lower FWAD and higher FWVF in both pAs and WM, particularly in frontal and temporal cortices. Elevated HbA1c and fasting glucose were also linked to widespread reductions in FWAD, primarily along periarterial pathways. Sleep quality (PSQI) showed a positive correlation with FWAD in the dorsal attention network and a negative correlation with periarterial FWVF in the visual network.

Cognitive performance (MoCA) positively correlated with pAs-FWAD within the dorsal attention, salience, auditory, and default mode networks, whereas no significant relationships were observed for WM-FWVF or pVs-ALPS.

### Glymphatic alterations associated with Alzheimer's pathology and clinical stages in ADNI

We examined group differences in glymphatic metrics across CN, MCI, and AD participants from the ADNI dataset (Fig. 2), with age, sex, years of education, and APOE genotype (allele ε4 vs. others) as covariates. pAs-FWAD was significantly lower in Aβ-positive compared to Aβ-negative individuals within both the precuneus (p = 0.038) and AD-signature regions (p = 0.003). pAs-FWVF in these same regions was significantly higher in the Aβ-positive group than the Aβ-negative (p = 0.008 in precuneus and 0.014 in AD-signature regions). Similarly, WM-FWVF was significantly higher in Aβ-positive participants within the precuneus (p = 0.041) and AD-signature regions (p = 0.009). However, the global pAs-FWAD, pAs-FWVF, and WM-FWVF showed no significant differences between Aβ-positive and Aβ-negative groups. The ALPS index showed no significant difference between Aβ groups, though a non-significant trend toward higher values in Aβ-positive individuals was observed.

When stratified by diagnostic group, pAs and WM-FWVF in the whole brain, precuneus and AD-signature regions showed significant group differences between all pairs of CN, MCI and AD groups (whole brain pAs/FWVF: p = 0.002/0.011; precuneus: p = 0.002/0.011; AD-signature: p = 0.003/0.009). pAs-FWAD in the whole brain, precuneus, and AD-signature regions exhibited significant differences observed between CN and MCI (whole brain: p = 0.006; precuneus: p = 0.002; AD-signature: p = 0.003). We observed significant differences in ALPS between CN and AD (p = 0.009) and between MCI and AD (p = 0.014) but not between CN and MCI (p = 0.738).

### Classification of Aβ positivity and clinical diagnosis

We next evaluated the ability of glymphatic metrics to classify Aβ-PET positivity using pairwise DeLong tests. The integrated glymphatic model combining all three components achieved the highest discriminative performance (AUC = 0.817 when including subjects with all stages of CN + MCI + AD, AUC = 0.823 for those with non-demented stages of CN + MCI only), significantly outperforming models using WM-only features (AUC = 0.633 for all and 0.626 for non-demented, ΔAUC = 0.184–0.197; p = 3.2×10^− 4^) and ALPS (AUC = 0.606 for all and 0.612 for non-demented, ΔAUC = 0.211; p = 1.3×10^−5^). A combined pAs + WM model (AUC = 0.802 for all, and 0.803 for non-demented) also performed significantly better than either WM alone (ΔAUC = 0.169–0.177; p = 1.6×10^− 2^) or ALPS (ΔAUC = 0.191–0.196; p = 2.7×10^− 4^). Among single-compartment models, pAs (AUC = 0.74 in all and 0.727 in non-demented) outperformed both WM (ΔAUC = 0.101–0.107; p = 0.031) and ALPS (ΔAUC = 0.115–0.134; p = 0.001). Although the integrated model achieved the numerically highest AUC, its improvement over pAs + WM (ΔAUC = 0.015–0.02; p > 0.1) or pAs alone (ΔAUC = 0.077–0.096; p = 0.06) did not reach statistical significance. These findings indicate that pAs features provide the most robust discriminative signal, while WM integration enhances stability, and full glymphatic integration yields only marginal gains.

For comparison with a PET biomarker, three combined features of the precuneus, AD-signature and whole brain tau-PET SUVR yielded AUCs of 0.812 (all participants) and 0.796 (non-demented), displaying significant difference in the discriminative performance between integrated glymphatic measures and established molecular imaging biomarkers.

In differentiating clinical diagnostic groups (CN vs. MCI + AD and CN vs. MCI; Fig. 3), the integrated glymphatic features again achieved the highest overall accuracy (AUC = 0.812–0.821), significantly outperforming both pVs-only (ΔAUC = 0.200–0.208; p = 3.1×10^− 4^) and WM-only (ΔAUC = 0.186–0.188; p = 8.0×10^− 5^) models. Among individual components, pAs features (AUC = 0.795–0.802) exhibited markedly higher discriminative ability than pVs (ΔAUC = 0.181–0.191; p = 3.7×10^− 4^) and WM (ΔAUC = 0.169; p = 0.04). Adding WM to pAs modestly improved performance (AUC = 0.787–0.789) but achieved a marginal significance (ΔAUC = 0.008–0.013; p = 0.05).

For comparison with AD pathological biomarkers, combined Aβ- and tau-PET SUVR measures achieved lower AUCs (CN vs. MCI + AD: Aβ = 0.669, tau = 0.743; CN vs. MCI: Aβ = 0.648, tau = 0.698) than the integrated glymphatic features (CN vs. MCI + AD: ΔAUC = 0.076; p = 0.031; CN vs. MCI: ΔAUC = 0.124; p = 0.009). Combining tau with Aβ did not substantially improve performance compared to tau only.

### Mediation analysis: blood pressure, glymphatic function, amyloid burden, and cognition

We conducted a serial mediation analysis to examine whether the relationship between elevated blood pressure, a key factor that dysregulates arterial pulsatility, and cognitive decline (MoCA) was mediated by glymphatic dysfunction and amyloid burden (Fig. 4). Diastolic blood pressure (BP) was positively associated with periarterial FWAD in the precuneus (a_1_ = 0.00002, p = 0.03), which, in turn, was negatively associated with Aβ SUVR (a_2_ = − 64.01, p < 0.05). Higher Aβ SUVR was associated with poorer cognitive performance (b = − 2.733, p = 0.02). The total effect of diastolic BP on MoCA was not significant (c = − 0.031, p = 0.39), and the direct effect after accounting for both mediators remained non-significant (c’ = − 0.039, p = 0.27). However, the indirect effect through both mediators was significant (0.0032, 95% CI, 0.0002, 0.008), indicating that elevated diastolic BP contributes to cognitive decline indirectly by reducing periarterial water mobility (lower FWAD) and subsequently promoting greater Aβ accumulation on PET.

## Discussion

In this study, we used multi-compartment glymphatic metrics derived from a bi-tensor model using multi-shell diffusion MRI to characterize glymphatic alterations across pAs, WM parenchyma, and pVs in two complementary cohorts. Our principal findings are fourfolds: first, measures reflecting increased pAs free-water volume and reduced pAs water mobility were significantly linked to aging, CMS factors, and cognition in healthy aging adults and were also observed in Aβ-positive and cognitively impaired participants, with early changes observed in the precuneus and AD-signature regions. Second, pVs diffusivity decline was associated with aging and was most marked at later clinical AD stages, suggesting a downstream parenchymal and efflux failure following early periarterial compromise. Third, integrated glymphatic metrics combining periarterial, WM, and perivenous glymphatic features classified Aβ-PET positivity, with performance comparable to that of tau PET and clinical diagnosis, and outperformed combined tau and amyloid PET measures. Finally, a serial mediation analysis implicated a directional association linking higher vascular risk factors, pAs glymphatic dysfunction, Aβ tissue accumulation, and cognitive decline, with a statistically significant indirect effect. Together, using multi-compartment diffusion MRI in two large cohorts, our results support a stage-dependent compartmental model of glymphatic dysfunction in human aging and AD.

### Impact of Aging and Cardiovascular Risk Factors on Glymphatic Function

Aging is a well-established contributor to glymphatic system decline, and our findings from pAs-FWVF, WM-FWVF, and pVs-ALPS measurements are consistent with this framework. These metrics revealed age-related deterioration in pAs, pVs, and WM microstructural integrity, supporting the notion that advancing age compromises all three compartments of glymphatic system.

Beyond aging, specific CMS risk factors demonstrated distinct associations with pAs functionality, a marker closely linked to arterial pulsatility^17,18^. In particular, reduced pAs-FWAD was strongly associated with elevated diastolic blood pressure and fasting glucose levels, suggesting that vascular stiffening and metabolic dysregulation jointly compromise the pulsatile forces driving periarterial CSF transport. Hypertension alters the hemodynamic profile of arterial pulsation in ways detrimental to perivascular flow. In animal models, two-photon microscopy has shown that CSF transport along periarterial channels is driven by rhythmic arterial wall motion, which becomes markedly attenuated under hypertensive conditions due to reduced arterial compliance despite increased pulsation amplitude^17^. A 7T MRI human study similarly revealed elevated microvascular volumetric pulsatility in deep WM among hypertensive and older adults^19^, consistent with the increased pulsation amplitude observed in animal models. Collectively, these findings suggest that hypertension amplifies local vascular stress yet disrupts the coordinated wall motion required for efficient glymphatic transport.

Chronic hyperglycemia, as seen in diabetes and poorly controlled glucose levels, may exacerbate glymphatic dysfunction through mechanisms such as microangiopathy, oxidative stress, and alterations in the perivascular basement membrane composition, all of which can impede interstitial solute clearance^20–22^. This interpretation aligns with animal studies demonstrating that experimental diabetes leads to perivascular AQP4 mislocalization and reduced glymphatic transport efficiency^22^.

Notably, the associations between fasting glucose, blood pressure, and pAs-FWAD were no longer significant after adjusting for antihypertensive and antidiabetic medication use. This attenuation implies that pharmacological control of vascular and metabolic risk factors may help preserve periarterial fluid mobility. Effective management of blood pressure and glucose has been shown to improve cerebrovascular compliance and reduce arterial stiffness^23^, which may in turn stabilize the mechanical driving forces underlying glymphatic transport.

Other CMS-related factors, including lower HDL cholesterol, elevated BMI, and higher triglyceride levels were associated with both lower pAs-FWAD and higher pAs- and WM-FWVF, but not with pVs-ALPS. These patterns suggest a combination of microvascular compromise and extracellular space expansion, possibly reflecting chronic low-grade inflammation and tissue remodeling. The convergence of these vascular–metabolic insults may not only hinder clearance but also promote amyloid accumulation and white matter damage over time^24^.

Taken together, our findings support the concept that both aging and CMS components exert additive and potentially synergistic effects on glymphatic function, with periarterial pathways appearing especially vulnerable to hemodynamic and metabolic stressors.

### Role of Sleep Disturbances in Glymphatic Dysfunction

Sleep disturbances critically affect glymphatic clearance and, consequently, AD-related neurodegeneration. In our study, poor sleep quality, indicated by high PSQI scores, was associated with pAs-FWAD in the frontoparietal network and FWVF in the visual network. Animal studies have robustly demonstrated that sleep deprivation impairs glymphatic clearance^25^, though human evidence remains limited. Recent work by Ma et. al. indicates that sleep quality directly influences glymphatic function and brain networks critical for visual memory in older adults^26^. Likewise, lighter sleep in middle-age and elderly individuals has been linked to an increased burden of enlarged perivascular spaces^27^. Aging exacerbates the adverse effects of poor sleep by impairing microglial and macrophage function, fostering a pro-inflammatory environment^28,29^, which may further impede glymphatic flow. Such chronic inflammation may lead to reactive astrogliosis resulting in AQP4 depolarization and reduced AQP4 expression, consequently impeding CSF–ISF exchange^30^.

### Glymphatic Dysfunction in AD Progression: Novel Associations between Compartments and AD Stages

Our compartment-based analysis revealed stage-dependent alterations, with pAs and WM changes emerging in early phases of AD, and pVs alterations becoming more pronounced in later stages. This pattern aligns with prior experimental and modeling studies in mice, which show that the pAs plays a disproportionately greater role in solute exchange between CSF and brain parenchyma compared with the pVs, owing to its greater abundance, larger caliber, and higher net CSF inflow^31,32^.

In early stages of AD, reduced pAs-FWAD, potentially influenced by aging, poor sleep, and CMS-related factors^18,33^, likely reflects diminished arterial pulsatility and other driving forces that propel glymphatic inflow^34^. Such impairments would slow the clearance of soluble waste products, including Aβ and tau, leading to their gradual accumulation in the interstitial space^3,35^. In contrast, alterations in pVs functionality become more prominent in later stages of disease. These changes likely represent downstream consequences of chronic glymphatic failure, characterized by neuroinflammation and elevated flow resistance from Aβ and tau deposition and extracellular water retention^36,37^.

Supporting this interpretation, previous studies have shown that cerebral amyloid angiopathy and Aβ accumulation in pAs can promote extracellular fluid buildup^38^, possibly through reactive astrogliosis^39^ and chronic inflammation^2^. Astrocytic dysfunction, particularly of aquaporin-4 (AQP4) channels on astrocyte endfeet, may further disrupt water and ion homeostasis, exacerbating clearance deficits^39,40^.

These stage-specific alterations provide a mechanistic explanation for the predictive patterns observed in our data. Because pAs dysfunction arises earlier, its metrics likely capture preclinical glymphatic deficits associated with initial amyloid accumulation, whereas pVs changes reflect later-stage pathology and vascular comorbidities. Integrating pAs, WM, and pVs features thus enables a comprehensive characterization of both early and late glymphatic failure. This multimodal approach achieved high accuracy in classifying individuals across the AD spectrum (CN, MCI and AD) and in predicting early Aβ accumulation—comparable to tau PET performance in non-demented individuals and superior to combined amyloid and tau PET features in differentiating MCI from CN. These results highlight the promise of DTI-based glymphatic markers as a cost-effective, noninvasive tool for staging and risk stratification in AD.

Furthermore, our regional mapping successfully localized alterations of pAs and WM function in the AD-signature and precuneus regions, highlighting their association with early disease progression. This regional mapping approach may also help delineate disease-specific glymphatic dysfunction patterns in other neurovascular or neurodegenerative disorders, such as cerebral small vessel disease or α-synucleinopathies. For instance, pAs abnormalities may cluster around cerebral microbleeds in small vessel disease or localize to the substantia nigra and basal ganglia in Parkinson’s disease, where α-synuclein aggregation contributes to neurovascular and autonomic dysregulation.

### Vascular–Glymphatic–Amyloid–Cognition Pathway

Our serial mediation analysis revealed a significant indirect pathway linking diastolic blood pressure to cognitive performance via pAs-FWAD and amyloid burden in the precuneus. This supports the concept that vascular risk factors—particularly elevated blood pressure—can impair periarterial function, which in turn accelerates amyloid deposition and its downstream neurotoxic effects. Notably, pAs FWAD reductions are consistent with early-stage glymphatic dysfunction, likely driven by diminished arterial pulsatility and other flow-driving forces, as described in our stage-dependent model. Although the total and direct effects of blood pressure on cognition were non-significant, the robust indirect pathway underscores the mechanistic relevance of vascular–glymphatic–amyloid interactions.

These results align with prior animal findings indicating that vascular dysfunction, particularly hypertension, contributes to impaired glymphatic clearance^41,42^, promoting early amyloid accumulation in regions such as the precuneus—a hub of the default mode network particularly vulnerable in AD^43,44^. Moreover, the mediation pattern suggests that interventions aimed at preserving or restoring pAs function (e.g., optimizing blood pressure control, improving sleep quality, and enhancing vascular pulsatility) may delay the onset of amyloid-related neurotoxicity and cognitive decline. Such strategies could offer a mechanistically targeted approach to risk reduction, complementing amyloid- or tau-directed therapies and potentially serving as early preventive measures in at-risk populations.

### Limitations and Future Directions

Diffusion MRI with high *b*-values reflects water mobility and free-water accumulation, providing an index of long-term glymphatic integrity rather than real-time CSF flow dynamics.

The pAs diffusivity metrics were derived from semi-automated segmentation; although sensitivity analyses using the largest pAs volumes were performed, the influence of segmentation variability on measurement accuracy remains to be fully clarified.

Potential confounders beyond age, sex, APOE genotype, and education—such as socioeconomic status, which is closely linked to sleep quality and cardiovascular health—were not included in this analysis. Moreover, the ADNI dataset lacks detailed sleep information, limiting the assessment of how sleep modulates glymphatic function in AD progression.

The spatial resolution of diffusion MRI (1.5–2 mm) in the HCP and ADNI datasets may introduce partial volume effects, potentially affecting the precision of pAs free-water volume and diffusivity estimates, particularly when the pAs diameter^45^ segmented on T1- or T2-weighted MRI falls below the diffusion voxel size. This limitation was mitigated by focusing on the four largest pAs in each ROI. We observed that the mean pAs diameters for all ROIs exceeded the resolution of DWI data. Furthermore, FWVF values were significantly higher in pAs than in WM, as expected (**Table S3**).

Finally, given the cross-sectional design, causal relationships cannot be inferred. Longitudinal studies incorporating advanced imaging (e.g., contrast-enhanced MRI) and mechanistic validation are warranted to delineate the temporal dynamics of glymphatic impairment. Future investigations should also test whether targeted interventions, such as sleep optimization or vascular health management, can restore glymphatic function and mitigate downstream neurodegeneration.

## Conclusion

This study identifies stage-dependent glymphatic alterations across aging and Alzheimer’s disease (AD) progression, revealing distinct compartmental contributions to disease evolution. In cognitively normal individuals, periarterial dysfunction was associated with vascular risk and sleep disturbances, whereas pAs and WM impairments preceded amyloid accumulation. In contrast, perivenous deficits emerged later, accompanying protein aggregation and cognitive decline. Regional mapping localized early pAs and WM alterations to regions vulnerable to cardiometabolic and sleep-related stress, underscoring their potential as sensitive, spatially specific biomarkers of preclinical disease. Moreover, the vascular–glymphatic–amyloid–cognition pathway analysis demonstrated that vascular health influences cognition indirectly through its effects on glymphatic function and amyloid burden. Collectively, these findings position the pAs as a critical locus of early vulnerability and a promising target for intervention. Strategies that improve cardiovascular health, enhance sleep quality, and restore glymphatic clearance may hold potential to delay amyloid pathology and preserve cognitive function.

## Methods

### Study participants

We analyzed two independent cohorts. The first cohort included 579 cognitively normal (CN) adults aged > 36 years from the HCP-Aging Lifespan Release 2.0^11,46^. Participants were recruited at Washington University in St. Louis, the University of Minnesota, Massachusetts General Hospital, and the University of California, Los Angeles. Exclusion criteria included the use of special educational services, MRI contraindications, history of major medical illness, head injury, endocrine or psychiatric disorders, and neurodevelopmental disorders.

The second dataset used in this study was obtained from the Alzheimer’s Disease Neuroimaging Initiative (ADNI) database (adni.loni.usc.edu). ADNI was launched in 2003 as a public–private partnership, led by Principal Investigator Michael W. Weiner, MD. The primary goal of ADNI is to test whether serial magnetic resonance imaging (MRI), positron emission tomography (PET), other biological markers, and clinical and neuropsychological assessments can be combined to measure the progression of mild cognitive impairment (MCI) and early Alzheimer’s disease (AD).

For this study, ADNI participants were included only if they had multi-shell DWI, Fluid Attenuated Inversion Recovery (FLAIR) MRI, T1-weighted MRI, amyloid and tau PET, and covariate data (age, sex, education, and APOE ε4 genotype). Because multi-shell DWI is available exclusively in ADNI phases 3 and 4, the sample was restricted to these phases to leverage multi-shell diffusion data for compartment-specific modeling.

AD dementia diagnosis followed NINCDS–ADRDA criteria^47^. MCI was diagnosed by a comprehensive evaluation of Mini-Mental State Examination (MMSE) scores (24–30), impaired delayed recall on the Wechsler Memory Scale Logical Memory II, Clinical Dementia Rating–Sum of Boxes (CDR-SB) ≥ 0.5, preserved daily functioning, and absence of dementia. Cognitively normal (CN) controls were defined as having MMSE ≥ 24 and CDR-SB = 0 or 0.5. The final ADNI sample comprised 10 AD dementia, 68 MCI, and 95 CN participants.

Both the ADNI and HCP-Aging studies were approved by institutional review boards of all participating centers, and written informed consent was obtained from all participants.

### MRI and PET imaging

HCP-A participants were scanned on a Siemens 3T Prisma system equipped with an 80 mT/m gradient coil and a Siemens 32-channel head coil. Diffusion-weighted images (DWI) were acquired using a single-shot spin-echo EPI sequence with the following parameters: TR = 4700 ms, TE = 64 ms, multiband factor = 2, GRAPPA R = 2, 6/8 partial Fourier, 6 b_0_ volumes, 45 diffusion directions with b = 1500 s/mm^2^, and 45 with b = 3000 s/mm^2^. A total of 90 slices were acquired with 1.5 mm thickness and no gap. The in-plane resolution was 1.5×1.5 mm, zero-filled to 0.75×0.75 mm, with a field of view (FOV) of 220 mm.

T1-weighted multi-echo MPRAGE scans were also acquired with TR = 2500 ms, inversion time (TI) = 1000 ms, TE = 1.8/3.6/5.4/7.2 ms, 4 echoes per line of k-space, voxel size = 0.8 mm isotropic, FOV = 256×240×166 mm, matrix = 320×300×208, and flip angle = 8°, following the Human Connectome Project–Aging protocol^46,48^.

In the ADNI-3 and ADNI-4 dataset, DWI imaging was performed with an echo planar imaging sequence using: 14 b_0_ volumes, six diffusion directions with b = 500 s/mm^2^, 56 with b = 1000 s/mm^2^, and 56 with b = 2000 s/mm^2^ (TE = 61.8–83.1 ms, TR = 9500–14,200 ms, matrix = 256×256, FOV = 350×350 mm, and slice thickness = 2.0 mm), resulting in voxel size of 2-mm isotropic. T1-weighted images were acquired using a spoiled gradient echo sequence with the following parameters: TR = 6.96–7.67 ms, TE = 2.83–3.17 ms, matrix = 256×256, FOV = 260×260 or 270×270 mm, and slice thickness = 1.2 mm. Fluid-attenuated inversion recovery (FLAIR) imaging was obtained with the following parameters: TE = 147.90–153.90 ms; TR = 11,000 ms; TI = 2250 ms; matrix = 256×256; FOV = 220×220 mm; and slice thickness = 5 mm. All the images were obtained from the ADNI database (http://adni.loni.usc.edu/).

The Aβ (florbetapir [^18^F–AV45] or florbetaben [FBB]) and tau (flortaucipir [^18^F–AV1451]) PET data were also available for the studied subjects. Aβ and tau SUVRs were calculated using the UC Berkeley amyloid and tau PET processing pipeline. Details of the processing protocol can be found in the open-source documentation (https://adni.loni.usc.edu/updated-uc-berkeley-amyloid-pet-methods/)

The overall pipeline for estimation of multimodal MRI features of glymphatic function is illustrated in **Supplementary Fig. S5**.

### Image processing and pAs segmentation, and false positive exclusion

Three-dimensional T1-weighted (T1w) volumetric images were preprocessed using the Montreal Neurological Institute (MNI) CIVET pipeline, which included brain extraction, bias-field correction, and tissue classification^49^. Binary brain tissue masks were generated by CIVET. After excluding dura mater, cerebral gray matter, cerebellum, and brainstem, cerebral WM was defined for pAs segmentation.

To enhance visibility, histogram equalization was applied to the T1w images, followed by non-local means denoising to reduce noise, as described in a prior study^50^. pAs were then segmented using a pre-trained nnUNet model as previously described^51^. All segmentations were visually inspected for quality by neurologists (J.H., K.W.K., J.W.S., and T.W.Y.) and trained raters (A.S.K. and J.C.), with manual corrections applied when necessary. For the HCP-A dataset, combined T1w and T2w images were used to improve visual inspection where needed.

To minimize contamination of pAs segmentation by WM hyperintensities (WMHs), falsely segmented WMHs and lacunes were excluded by evaluating morphology (linear vs. round), size, clustering, and anatomical location. In the ADNI dataset, available FLAIR images, which optimally delineate WMHs, were additionally used. WMHs were segmented from multimodal T1w and FLAIR images using the U-Net with highlighted foreground technique^52^. These FLAIR-derived WMH masks were then non-linearly registered to the corresponding T1w images, and WMH voxels were removed from the pAs annotations to ensure robust segmentation.

For analyses of CMS effects on glymphatic function in cognitively normal participants, pAs volumes segmented from HCP-A were mapped onto nine functional regions of interest (ROIs). These ROIs were defined on the cortical surface using the Automated Anatomical Labeling (AAL) template, following prior work^53^, and included the frontoparietal network, dorsal attention, ventral attention/language, sensorimotor, default mode network, auditory, visual, salience, and limbic network.

For analyses of AD progression, ROIs corresponding to the AD-signature, including bilateral hippocampi, temporal poles, and entorhinal, inferior temporal, superior frontal, inferior parietal, and anterior cingulate cortices, were also defined using the AAL template. The precuneus, implicated in early AD pathology^43,44^, was separately delineated for targeted analyses.

ROIs were propagated into adjacent superficial white matter using *k*-nearest neighbors (k-NN) clustering, constrained to a depth of 5 mm from the gray matter–WM boundary using Laplacian distance mapping (**Supplementary Fig. S6**). A pAs volume was assigned to an ROI if it overlapped with the ROI mask.

### Diffusion-weighted MRI-based glymphatic metrics: FWVF, FWAD, and ALPS index calculation

Multi-shell diffusion-weighted imaging (DWI) data were corrected for head motion, eddy current distortions, and EPI-related susceptibility artifacts using FSL’s EDDY^54^. Unlike conventional free-water elimination DTI (FWE-DTI)^55^, which constrains the free-water compartment to be isotropic, the Tissue–Tensor Imaging (TTI) framework^16^ accounts for directional diffusion within perivascular and extracellular fluid spaces. This enables estimation of fluid-compartment diffusivity measures (FWAD, FWRD, FWMD, FWFA) alongside tissue-compartment metrics (tAD, tRD, tMD, tFA), where fAD reflects the axial diffusivity of the fluid tensor and is interpreted as an indicator of directionality in perivascular fluid movement^56^. Detailed methods are described in the **Supplementary Methods**.

Individual b_0_ images were linearly and then nonlinearly registered to each participant’s T1-weighted (T1w) space using ANTs with a mutual information similarity metric^57^. The resulting DWI-derived maps were subsequently warped to each subject’s T1w space using the deformation fields from this registration, aligning DTI-based metrics to the anatomical space where pAs annotations and the functional network ROIs and AD-related ROIs were defined (**Supplementary Fig. S5**).

This framework leveraged the anatomical role of pAs as cerebrospinal fluid (CSF)–filled conduits. Within each segmented pAs, free-water axial diffusivity (FWAD) was quantified as an index of fluid mobility, whereas free-water volume fraction (FWVF) was calculated to capture local CSF accumulation that may hinder fluid transport. FWAD and FWVF values were averaged across voxels within each pAs mask in T1w space. For complementary validation, FWFA, FWMD, and FWRD were also computed within the same pAs regions. Validation results are presented in **Supplementary Fig. S3**.

To assess free-water burden in the WM parenchyma, all WMH and pAs voxels were excluded from the WM mask. The mean FWVF of the remaining WM voxels within each ROI was then calculated and defined as the ROI’s WM-FWVF. Finally, interstitial fluid efflux along pVs was evaluated using the DTI-ALPS, as described in **Supplementary Methods**.

Because many pAs detected on high-resolution T1w MRI have diameters smaller than the effective DWI voxel size, diffusion measures within pAs masks may be affected by partial volume contamination. To mitigate this, we restricted analyses to the largest pAs within each ROI, determining the optimal number (*n*) that maximized measurement stability and sensitivity to clinical variables (**Supplementary Fig. S1**). The summed absolute t-values from correlations of FWAD and FWVF with age, PSQI, and CMS factors (adjusted for age, sex, and education) were highest when four pAs per ROI were included, indicating an optimal trade-off between sensitivity and sample retention. At this threshold, subject inclusion remained high (95% at four pAs vs. 87% at six pAs, particularly within the auditory network). Therefore, the four largest pAs per ROI were used for all subsequent analyses.

### Cognitive assessments

At the time of HCP-A recruitment, participants were in good health, without a diagnosed history of neurologic or major psychiatric disorder, symptomatic stroke or dementia. The self-reported Pittsburgh Sleep Quality Index (PSQI) questionnaire and Montreal Cognitive Assessment (MoCA) scores were included in this study.

For participants from the ADNI cohort, global cognitive function was assessed using both Mini-Mental State Examination (MMSE) and Montreal Cognitive Assessment (MoCA)^58^.

### CMS profiles and AD biomarkers

The HCP-A metadata provided comprehensive CMS profiles, including height, weight, blood pressure, fasting glucose, and lipid biomarkers. BMI was calculated as weight (kg) divided by height squared (m^2^), based on measurements obtained during participant interviews. Systolic and diastolic blood pressures were recorded with participants seated and mean arterial pressure (MAP) was derived as: MAP = (2 × diastolic blood pressure + systolic blood pressure) / 3. Metabolic biomarkers, including high-density lipoprotein (HDL), hemoglobin A1c (HbA1c), plasma glucose, triglycerides, and total cholesterol, were quantified from fasting blood samples.

The ADNI dataset provided a small number of cardiometabolic measures, including height, weight, and systolic and diastolic blood pressures, allowing BMI and MAP to be calculated using the same formulas applied in HCP-A. In addition to these cardiometabolic variables, ADNI participants were also characterized by Aβ status (positive or negative) determined by amyloid PET^59^, enabling subgroup comparisons and evaluation of glymphatic alterations in relation to AD pathology.

### Statistical analysis

All statistical analyses were conducted using Python (v3.9) and relevant packages including statsmodels and scipy.

To examine associations between glymphatic fluid metrics and either age or CMS in the HCP cohort, linear regression models were fitted for each metric separately. Independent variables included age, BMI, systolic and diastolic blood pressure, mean arterial pressure (MAP), HDL, HbA1c, triglycerides, and glucose. Age and sex were adjusted as covariates (only sex was included for correlations with age). Regression coefficients (standardized β-values) and p-values were extracted and visualized in scatterplots. False discovery rate (FDR) correction using the Benjamini-Hochberg procedure was applied across multiple comparisons within each anatomical compartment to control for type I error. Significance was defined as FDR-corrected p < 0.05, and shaded areas in plots represent 95% confidence intervals. Variables with non-significant associations were labeled as “NS”.

For the ADNI dataset, group-level differences in glymphatic dysfunction were examined using analysis of covariance (ANCOVA) across diagnostic groups (CN, MCI, and AD) and across amyloid status groups (A− and A+), adjusting for age, sex, APOE genotype (ε4 vs. non-carrier), and years of education. Post hoc pairwise comparisons were conducted using Tukey’s HSD test. Violin plots depict the distribution of each metric within groups, and statistically significant differences are marked with asterisks (*p < 0.05, **p < 0.01, ***p < 0.001). For features that did not conform to a normal distribution, nonparametric rank-ANCOVA and Wilcoxon rank-sum tests were applied for post hoc comparisons.

To evaluate the discriminative ability of glymphatic MRI metrics for Aβ-PET positivity and clinical diagnosis, supervised learning models were trained using a set of linear and nonlinear classifiers, including support vector machines (SVMs; linear and radial basis function kernels), extreme gradient boosting (XGBoost), and generalized linear models (GLMs). Input features were defined from three glymphatic compartments: (1) pAs features, including FWVF and FWAD within the precuneus, AD-signature, and whole brain; (2) WM features, including FWVF within the precuneus, AD-signature, and whole brain; and (3) the pVs feature, represented by the DTI-ALPS index. For molecular imaging biomarkers, Aβ-PET features included SUVRs in the precuneus, AD-signature, and whole brain, and tau-PET features included SUVRs in the same regions.

All models were trained and evaluated using stratified 10-fold cross-validation (number of splits = 10, random state = 42). Classification performance was quantified using the area under the receiver operating characteristic curve (AUC). Pairwise DeLong tests were conducted to assess statistical differences in AUC values between models, and corresponding AUCs are reported in Fig. 3. Detailed performance results for all classifiers are provided in **Table S4** – **S6**.

Mediation analyses were performed using a standard three-path model to test indirect effects (a × b) of age or CMS variables on outcome measures (MoCA, or MMSE) through glymphatic fluid metrics and Aβ-PET SUVR as mediators. Path coefficients (β), indirect effect estimates, and bias-corrected 95% confidence intervals were reported. Significance of indirect paths was determined via bootstrap resampling (n = 5,000). All variables were normalized to ensure comparability across predictors, and the significance of indirect effects was determined based on the bootstrapped confidence intervals that indirect effects are considered significant if the CI does not include zero.

## Supplementary Material

This is a list of supplementary files associated with this preprint. Click to download.

• Supplementarynpjsubmit.docx

## Figures and Tables

**Table 1:** Characteristics of HCP-Aging participants

Age (years)	N = 546
	58.61 (14.08)
Women, n(%)	312 (57.14)
BMI (kg/m^2^)^a^	27.10 (4.84)
Systolic (mmHg)^b^	129.05 (16.42)
Diastolic (mmHg)^b^	79.29 (10.45)
Mean arterial pressure (mmHg)^b^	95.00 (14.34)
High density lipoprotein (mg/dL)	60.05 (16.47)
Hemoglobin A1c (%)	5.36 (0.53)
Total cholesterol (mg/dL)	198.69 (36.41)
Triglycerides (mg/dL)	108.41 (68.34)
Fasting-glucose (mg/dL)	101.91 (17.94)
PSQI^c^	4.68 (2.69)
MoCA	26.40 (2.55)

Data are presented as mean (s.d.) or number (percentage).

a BMI was missing for two subjects.

b Blood pressure was missing for five subjects.

c PSQI was missing for one subject.

**Table 2:** Characteristics of ADNI participants

Age, years	All (n = 173)	CN (n = 95)	MCI (n = 68)	AD (n = 10)
	76.49 (7.33)	76.40 (7.48)	76.44 (7.00)	77.70 (8.60)
Women (%)	78 (45.1%)	45 (47.4)	28 (41.2)	5 (50.0)
APOE-ε4 carriers, n (%)^a^	54 (42.5)	24 (36.9)	24 (43.6)	6 (85.7)
Education, years^b^	16.73(2.31)	16.99 (2.33)	16.35 (2.65)	16.90 (2.01)
MMSE^c^	27.90 (2.68)	28.81 (1.32)	27.26 (2.89)	23.50 (4.88)
MoCA^d^	24.45 (4.15)	26.06 (2.45)	22.75 (4.86)	17.86 (3.13)
Aβ-PET measures	n = 158	n = 92	n = 59	n = 7
Amyloid positive (n, %)	0.45 (0.50)	0.33 (0.47)	0.58 (0.50)	1.00 (0.00)
Aβ SUVR (whole brain)	1.20 (0.27)	1.12 (0.23)	1.27 (0.28)	1.49 (0.34)
Aβ SUVR (precuneus)	1.29 (0.32)	1.21 (0.28)	1.37 (0.32)	1.59 (0.37)
Aβ SUVR (AD-Signature)	1.16 (0.27)	1.08 (0.23)	1.24 (0.27)	1.45 (0.36)
Tau-PET measures	n = 160	n = 90	n = 62	n = 8
Tau SUVR (whole brain)	1.19 (0.23)	1.13 (0.12)	1.23 (0.29)	1.48 (0.38)
Tau SUVR (precuneus)	1.19 (0.23)	1.13 (0.12)	1.23 (0.29)	1.48 (0.38)
Tau SUVR (AD-Signature)	1.32 (0.32)	1.21 (0.13)	1.40 (0.37)	1.96 (0.49)
BMI^e^	28.23 (11.95)	30.02 (15.89)	26.22 (3.88)	26.69 (6.97)
Systolic Blood Pressure^f^	136.91 (18.77)	135.97 (17.42)	138.03 (19.98)	138.00 (23.67)
Diastolic Blood Pressure^f^	73.53 (9.60)	73.65 (9.00)	73.15 (10.25)	75.00 (11.21)

Data are presented as mean (s.d.) or number (percentage). Aβ-PET positivity was determined as composite AV45 SUVR > 0.1.11 or FBB SUVR > 1.478.

a APOE-ε4 was missing for 30 subjects in CN, 13 in MCI, and 3 in AD.

b Education was missing for five subjects in CN and two in MCI.

c MoCA was missing for two subjects in CN, seven subjects in MCI and three in AD.

d MMSE was missing for two

p articipants in MCI.

e BMI was missing for two subjects in AD, 30 in CN and 17 in MCI.

f Blood pressures were missing for two subjects in CN.

## Data Availability

The data that support the findings of this study are available within the article and its supplementary information. Imaging data were obtained from publicly available resources, including the Alzheimer’s Disease Neuroimaging Initiative (ADNI; http://adni.loni.usc.edu/ ) and the Human Connectome Project via the NIH NIMH Data Archive (https://nda.nih.gov/ccf ). Details of ADNI SUVR PET processing are available at https://adni.loni.usc.edu/updated-uc-berkeley-amyloid-pet-methods/ . Region-of-interest (ROI) templates are available at https://github.com/Yaqiongchai/ROI-template. Binary periarterial space segmentation masks are available from the corresponding author upon reasonable request.
